# Long-Term Conservation for the Safeguard of *Abies nebrodensis*: An Endemic and Endangered Species of Sicily

**DOI:** 10.3390/plants13121682

**Published:** 2024-06-18

**Authors:** Carla Benelli, Waed Tarraf, Tolga İzgü, Monica Anichini, Cecilia Faraloni, Maria Cristina Salvatici, Nourhene Jouini, Maria Antonietta Germanà, Roberto Danti, Maurizio Lambardi

**Affiliations:** 1 Institute for BioEconomy (IBE), National Research Council (CNR), Via Madonna del Piano 10, 50019 Sesto Fiorentino, Florence, Italy; carla.benelli@ibe.cnr.it (C.B.); tolga.izgu@ibe.cnr.it (T.İ.); monica.anichini@ibe.cnr.it (M.A.); cecilia.faraloni@ibe.cnr.it (C.F.); maurizio.lambardi@ibe.cnr.it (M.L.); 2 Institute of Chemistry of Organometallic Compounds (ICCOM)-Electron Microscopy Centre (Ce.M.E.), National Research Council (CNR), Via Madonna del Piano 10, 50019 Sesto Fiorentino, Florence, Italy; salvatici@ceme.fi.cnr.it; 3 Department of Agricultural, Food and Forestry Sciences, University of Palermo, 90128 Palermo, Italy; nourhene.jouini@unipa.it (N.J.); mariaantonietta.germana@unipa.it (M.A.G.); 4 Institute for Sustainable Plant Protection (IPSP), National Research Council (CNR), Via Madonna del Piano 10, 50019 Sesto Fiorentino, Florence, Italy; roberto.danti@ipsp.cnr.it

**Keywords:** Sicilian fir, cryopreservation, cryobank, pollen, zygotic embryos, embryogenic callus

## Abstract

The combined approaches between ex situ and in situ conservation are of great importance for threatened species in urgent need of protection. This study aims to develop concrete actions to preserve the relic of 30 adult trees of the Sicilian fir (*Abies nebrodensis*) from extinction using long-term germplasm conservation in liquid nitrogen (LN, −196 °C). Pollen grains were collected, and their moisture content (MC) was measured. Then, viability (2,3,5-tryphenyl tetrazolium chloride, TTC), in vitro germinability, and enzymatic antioxidant activity (ascorbate peroxidase, APX; catalase, CAT) were evaluated before and after cryopreservation. Seeds collected from mature cones underwent X-ray analysis, and only full seeds were used to excise the zygotic embryos (ZEs) for cryopreservation. The MC percentage of ZEs was determined, and then they were plunged in LN with (+PVS2) or without (−PVS2) Plant Vitrification Solution 2; untreated ZEs were used as a control. Viability (TTC test) and in vitro germination were assessed for all ZEs (+PVS2, −PVS2, and control). Embryogenic callus (EC) lines obtained from mature ZEs were cryopreserved applying the ‘encapsulation-dehydration’ technique. This study has allowed, after optimizing cryopreservation protocols for pollen, ZEs, and EC of *A. nebrodensis*, to establish the first cryobank of this endangered species in Polizzi Generosa (Palermo, Italy), inside the ‘Madonie Regional Park’. The strategy developed for Sicilian fir conservation will pave the way for similar initiatives for other critically endangered conifer species.

## 1. Introduction

*Abies nebrodensis*, commonly known as the Sicilian fir, is a critically endangered conifer species endemic to the Madonie Regional Park in the north of Sicily. This species is subject to significant threats caused by genetic erosion, fragmentation, poor natural regeneration, and a high rate of empty seeds leading to low seed germination. The remaining natural population is alarmingly small, consisting of only 30 adult trees, underscoring the urgency for effective conservation strategies [1,2,3]. According to the International Union for Conservation of Nature (IUCN), *A. nebrodensis* is classified as CR-D in the Red List of Endangered Species (https://top50.iucn-mpsg.org/species/1; accessed on 12 March 2024).

Historical exploitation and habitat degradation have contributed to the species decline, with its wood once highly valued for construction due to its elasticity and resistance. The past few decades have seen various projects aimed at both in situ and ex situ conservation, including experimental plantations and protective measures like fencing to support natural regeneration. Studies have highlighted *A. nebrodensis* as a species highly variable in seed production and the vital role of embryo presence in seeds for germination [4]. In addition, this species is characterized by a very limited natural regeneration due to the high rate of empty seeds, slow growth of seedlings, rocky soils, and grazing by wild herbivores.

**Figure 1:**
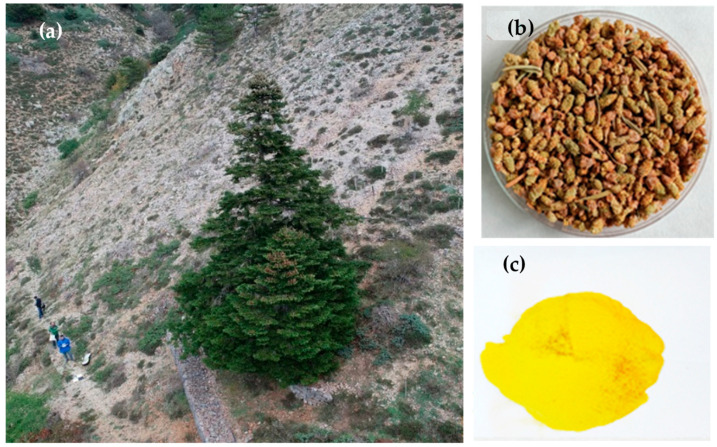
*Abies nebrodensis* tree (**a**); male cones (**b**); pollen (**c**).

The LIFE4FIR project (http://www.life4fir.com/it/), started in 2019, aims to develop an effective strategy to improve the conservation status of *A. nebrodensis* (Figure 1a) through a comprehensive approach that includes different actions: protecting the relic population, increasing genetic diversity by controlled cross-pollination, breeding selected outbred seedlings, and reforesting with selected seedlings to create new cores of the species within the Madonie Regional Park [1]. The project strategy is also based on ex situ conservation strategies, like establishing a seed bank and a cryobank for long-term germplasm conservation. Among the conservation techniques, cryopreservation stands out as a particularly promising method for the safe, long-term maintenance of *A. nebrodensis*.

The current conservation efforts greatly focus on biotechnological approaches, and cryopreservation has emerged as a crucial tool for the long-term maintenance of germplasm [5,6], allowing for the storage of plant material at ultra-low temperatures, effectively halting any enzymatic or chemical activity that could damage the biological material [7]. Under these conditions, any enzymatic or chemical activity is stopped, preventing structural and physiological damage to the stored biological material. This method allows for the indefinite storage of valuable plant material, making it a widely used tool for ex situ conservation of a variety of crops, fruit, and forest trees [8,9,10,11]. At present, plant cryopreservation techniques are available for storage of explants derived from in vivo and in vitro conditions [6,7,11], such as meristem and shoot tips, root tips, callus, pollen, seeds, cyanobacteria, algae, bryophytes, ferns, suspension cultures, embryogenic cultures of both zygotic and somatic embryos, and dormant buds [11,12,13,14,15]. 

This biotechnological approach for long-term conservation represents a safe and cost-effective tool where samples are stored in small volumes, protected from contamination, and without the need for laborious management. It should be noted that using desiccation, vitrification, and encapsulation-based methods for the cryopreservation of in vitro-derived shoot tips, several cryobanks of different economically important crops were established [16,17,18,19,20]. Similar efforts have been directed at the cryopreservation of some endangered species [21,22]. For example, several strategies were developed for threatened plants such as lilies, orchids, and redwood [23] as well as for the conservation of seeds and shoot tips of Canadian cherry birch [24]. 

In addition to shoot tips, several other explant types have been used in cryopreservation, such as pollen, zygotic embryos (ZEs), and embryogenic callus (EC). Particularly, pollen conservation is an important tool for the maintenance of plant genetic resources and can promote improved efficiency in breeding programs as well as germplasm conservation and exchange [25]. Cryopreservation of embryogenic tissue enables large-scale propagation and preservation of forestry resources [26]. Zygotic embryos, embryonic axes, and somatic embryos of different temperate and tropical species, including crops, fruit, and forest trees, have been effectively cryopreserved [27]. 

Within the genus *Abies*, the species subjected to cryopreservation include *A. cephalonica* L. [28,29], *A. nordmanniana* [30,31], *A. alba* [32], and some fir hybrids [33]. Currently, methodologies of conifer breeding integrate cryopreservation with somatic embryogenesis, extensively evaluated for *A. alba* [32].

For the above reasons, this study was focused on finding the optimal protocols for cryopreservation of pollen, ZEs, and EC to establish a cryobank for the critically endangered *A. nebrodensis.*

## 2. Materials and Methods

### 2.1. Plant Material 

Mature cones of *Abies nebrodensis* trees were collected during October 2020 in their natural habitat, located in the north-west of Sicily, in the Madonie Regional Park, Palermo (Italy), considering all the relevant guidelines and regulations to avoid any damage to the relict trees. Seeds extracted from a large part of the 30 residual trees were packed in sealable paper bags, signed, and transferred to the laboratory of the CNR-IBE, where they were stored at 4 °C until further use. In May 2022, the male cones were gently removed from adult trees. It was not possible to sample all 30 trees due to either a lack of seed production from some trees (only 24 are mature) or difficulties in collecting cones from trees located in impervious sites.

### 2.2. X-ray Analysis 

To select full seeds (i.e., seeds containing well-formed embryos), all seeds extracted from mature cones from trees numbers 6, 8, 10, 12, 13, 19, 21, 22, and 27 were X-ray radiographed through the X-ray apparatus “Gilardoni radio light” (Lecco, Italy) [2] set up as follows: 25 kV, 3 mA (soft X-rays), a focus-film distance of 45 cm, and a 2-min time of exposure. Seeds from each tree were placed in plastic square well plates (20 × 20 cm), 100 at a time, each seed in a separate well. The seeds were in direct contact with a Carestream X-ray film during the exposure. After X-ray application, the films were washed in development solution (4 min) and then in fixation solution (3 min) on a shaker (20 rpm). The films were washed under tap water (3–5 s) before further examination by the film viewer screen. 

### 2.3. Procedures for the Conservation in Liquid Nitrogen (LN, −196 °C)

#### 2.3.1. Pollen

Male cones were collected in mid-May 2022 from trees N° 1, 2, 6, 7, 8, 9, 10, 11, 12, 13, 14, 15, 16, 17, 18, 19, 21, 22, 23, 24, 27, and 29. After extraction from cones, the pollen was cleaned and sieved to remove all the impurities (Figure 1b,c) and maintained in paper bags at room temperature for 2 days. The moisture content (MC) of fresh pollen from different trees was measured using the moisture analyzer (Mettler-Toledo AG, Laboratory & Weighing Technologies, Greifensee, Switzerland). Measurements of MC were performed with 0.1 g of pollen per replicate and repeated three times for each tree. 

Morphological pollen observations were carried out at the CEME-Centro di Microscopie Elettroniche “Laura Bonzi”-CNR Research Area (Florence, Italy), using a Gaia 3 (Tescan s.r.o., Brno, Czech Republic) FIB-SEM (Focused Ion Beam-Scanning Electron Microscope) electron beam for scanning electron microscope (SEM) imaging with a voltage of 2 kV, operating in high vacuum mode, and a secondary electron (in-beam SE) detector. Samples were deposited on a stub and then coated with an ultrathin coating of gold. Pollen grains were photographed, and SEM measurements of the polar axis (P) were performed. At least 70 fresh pollen grains were examined to ensure a complete morphological analysis. Furthermore, the pollen observations were complemented by a stereomicroscope (Zeiss Stemi 2000 C, Jena, Germany) and an optical microscope (Leica DM-500, Heerbrugg, Switzerland) for counting viable and germinated pollen grains.

##### Viability Test

To evaluate the viability of pollen before and after LN immersion, the 2,3,5-tryphenyl tetrazolium chloride (TTC) test was used. A TTC 1% solution (purity 99%, Sigma-Aldich, Buchs, Switzerland) was prepared by adding 200 mg of TTC and 12 g of sucrose in 20 mL of distilled water. Two drops of this mixture were dropped on a microscope slide; the pollen was dusted over it, covered with a coverslip, and kept at room temperature for 24–48 h in the dark [34]. Following incubation, 400 pollen grains were randomly counted under the optical microscope (Leica DM-500). Pollen grains stained red were categorized as “viable”, whereas colorless-stained pollen was “non-viable”. The estimation of pollen vitality was expressed as the percentage of stained grains in the total number of grains counted. The TTC test was performed in two replicates for each tree. Three microscopic field views were observed per replicate, containing a minimum of 300 pollen grains/replication.

##### In Vitro Germinability Test 

The assessment of in vitro pollen germinability, both prior to and after cryopreservation, was carried out by placing pollen grains on a semisolid medium and evaluating the elongation of the pollen tube. After 48 h of incubation, the pollen tube that achieved a length at least three times the diameter of the pollen grain was considered germinated [35]. The composition of the germination medium consisted of boric acid (50 mg L^−1^), sucrose (15 g L^−1^), and plant agar (6 g L^−1^; Duchefa Biochemie, Haarlem, The Netherlands). The pollen was maintained in the dark at 25 °C as an optimal temperature for in vitro germination assays of most species [36]. For each plant, two replicates were conducted, and within each replicate, three microscopic fields were randomly selected for observation (at least 300 grains/replicate). The percentage of germination represented the number of germinated pollen grains in relation to the total number of grains counted. The number of germinated pollen grains was counted using a Leica DM-500 optical microscope.

##### Pollen Catalase and Ascorbate Peroxidase 

The determination of catalase (CAT) and ascorbate peroxidase (APX) activity was carried out for pollen samples of trees 6, 8, 9, 11, 13, 14, 22, 24, and 27, according to Ren et al. [37]. For both CAT and APX activity measurement, three replicates of 0.03 g pollen for each tree, before and after immersion in LN, were used for homogenization and extraction. For CAT activity, the absorbance, after the addition of 30% H_2_O_2_, was measured at a wavelength of 240 nm. For APX activity, the absorbance, after the addition of 30% H_2_O_2_, was measured at a wavelength of 290 nm. Each analysis was repeated three times. The results are expressed as U/g protein.

##### Immersion, Storage in Liquid Nitrogen, and Recovery of Pollen

**Figure 2:**
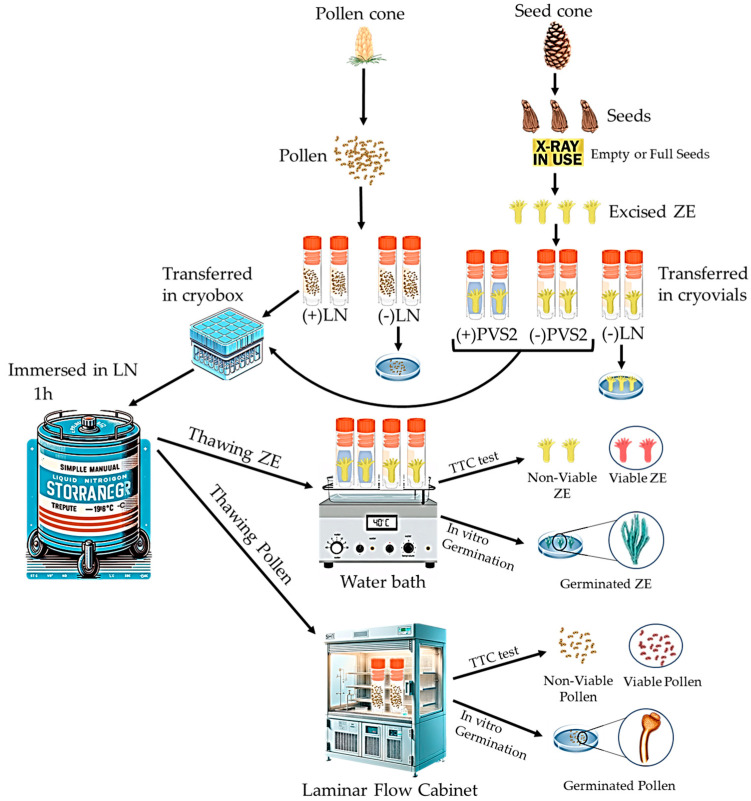
Steps of cryopreservation process for pollen and zygotic embryos (ZE) from *A. nebrodensis.* TTC: tryphenyl tetrazolium chloride; PVS2: Plant Vitrification Solution 2; LN: liquid nitrogen.

Samples of pollen were transferred in 2-mL cryovials. For each tree, two cryovials (0.30 g/cryovial) were immersed directly into LN. After storage in LN, for at least 1 h, the cryovials containing the pollen were thawed under a laminar flow cabinet for 2 h at room temperature. Subsequently, following the procedure described above, the viability (TTC) and in vitro germinability of cryopreserved pollen (Figure 2) were tested. 

#### 2.3.2. Excised Zygotic Embryos 

Under laminar flow, X-rayed seeds containing mature embryos were treated with 70% EtOH (5 min), rinsed with sterile distilled water 3 times, treated with sodium hypochlorite (20% *v*/*v*) with a few drops of Tween 20 solution (20 min), and rinsed again 3 times with sterile distilled water. Finally, seeds were imbibed in water for 48 h under sterile conditions and then opened to excise the ZEs. 

##### Viability (TTC Test) and In Vitro Germination Test of ZEs 

The viability of ZEs was determined by the TTC. Briefly, ZEs were soaked in TTC solution 0.1% (*w*/*v*) in 50 mM Tris-HCl buffer (pH 7.6) for 24 h, in total darkness at 30 °C. After staining, the ZEs were placed on moist filter paper to observe their viability. The red color of embryonic tissues was the main indicator of ZEs viability. 

For the germination test, before and after the LN, excised ZEs were cultured in vitro on hormone-free Murashige and Skoog (MS; Sigma-Aldrich, St. Louis, MO, USA) [38] medium (MS-HF), containing sucrose (20 g L^−1^) and agar (7 g L^−1^) at pH 5.8. All ZEs were maintained at 24 °C under a 16-h photoperiod (60 μmol m^−2^s^1^ of photosynthetically active radiation). After 3 weeks of culture, the germination rate was evaluated. 

##### Immersion in LN and Recovery of ZEs

Prior to the immersion in LN, the MC of ZEs (approx. 15 ZEs = 0.1 g/tree) was determined under the sterile laminar airflow following the method of Ayala et al. [39]. From each tree, a total number of 108 ZEs was divided into three groups; each group included three replicates: (1) submerged in Plant Vitrification Solution 2 (+PVS2; 30% glycerol, 15% ethylene glycol, 15% dimethyl sulfoxide in MS, and 0.4 M sucrose [40]), (2) without Plant Vitrification Solution 2 treatment (−PVS2), and (3) untreated control (neither PVS2 nor LN). The cryovial (2 mL) containing 4 ZEs from the +PVS2 and −PVS2 groups were immersed in LN for at least 1 h. Thereafter, the cryovials were taken out of the LN and thawed in a water bath (40 °C) for 1 min. Under laminar flow, cryopreserved ZEs were washed with liquid MS medium containing 1.2 M sucrose (washing solution) for 20 min. All ZEs cryopreserved (+PVS2 and −PVS2) or not cryopreserved (control) were subjected to viability and in vitro germination tests (Figure 2), as described above. Both tests were carried out with three replicates per tree (6 zygotic embryos/replicate). 

#### 2.3.3. Embryogenic Callus 

The EC was obtained following a protocol developed by Jouini et al. [2]. Briefly, ECs obtained from ZEs were developed and subcultured every 4 weeks on Schenk and Hildebrandt (SH; Sigma-Aldrich, St. Louis, MO, USA) [41] media supplemented with 1 mg L^−1^ 6-benzyladenine (BA; Sigma-Aldrich, St. Louis, MO, USA), 1 g L^−1^ casein (Sigma-Aldrich, St. Louis, MO, USA), 500 mg L^−1^ glutamine (Duchefa Biochemie, Haarlem, The Netherlands), 20 g L^−1^ sucrose, and 7 g L^−1^ agar at 24 °C in dark conditions. After eight subcultures, the EC cryopreservation was carried out using the encapsulation-dehydration technique. Portions of EC obtained from the ZE of tree N°10 were encapsulated in Ca-Alginate beads as described by Standardi and Micheli [42]. Then, the beads were transferred into sterilized filter paper inside the Petri dishes (60 Ø mm) and placed inside a glass jar (500 cc) containing 150 g of silica gel previously sterilized in an oven at 105 °C for 16 h. The jars were sealed with tape, and the beads were exposed to silica gel at room temperature for 1, 2, 3, 4, and 5 h. A control treatment of encapsulated ECs without desiccation was also included. The desiccated beads were placed in 2 mL cryovials (5 per each), replicated three times, and plunged directly into LN for at least 1 h. Thereafter, cryopreserved encapsulated ECs in cryovials were thawed in a water bath at 40 °C for 2 min and treated with a washing solution for 20 min. Beads were placed on fresh SH regrowth medium, supplemented with 1 mg L^−1^ BA, 1 g L^−1^ casein, 500 mg L^−1^ glutamine, 20 g L^−1^ sucrose, and 7 g L^−1^ agar. The regrowth ability of encapsulated ECs was determined when the callus broke through the gel of the bead. The same protocol was applied for the other ECs obtained from trees N° 7, 8, 21, and 22. 

### 2.4. Statistical Analysis

The primary cause of the variation in the amount of zygotic embryos and pollen collected from *A. nebrodensis* trees depends on the production of trees, which was extremely variable from one year to the next. All cryopreservation experiments followed the randomized block trial design. The moisture content of pollen, the viability and germination of pollen, and ZEs, both before and after cryopreservation, were expressed as percentages. All percentages of data were subjected to an arcsine transformation. Means were differentiated through analysis of variance (ANOVA), followed by the least significant difference (LSD) post-hoc test to evaluate the differences in germination and viability of pollen and ZEs, and CAT and APX data analysis activities within *A. nebrodensis* trees. For the moisture content of the pollen, Duncan’s multiple range test was used. Data analysis was conducted using JMP^®^ software (SAS Institute, Cary, NC, USA) version 5.00, with the significance level at *p* ≤ 0.001. The Pearson correlation analysis between the germination of pollen and its viability, as well as the germination of ZEs and its viability, was computed using the corrplot package in the R programming language (version 4.3.1). 

## 3. Results and Discussion 

The *A. nebrodensis* species is affected by a very low seed germination rate, mainly depending on a large number of empty seeds [4]. Moreover, Sicilian fir is characterized by abundant seed production every 3–4 years, similar to other conifer species [43]. For this reason, the quantity of explants and the number of trees analyzed varied in the current study.

### 3.1. Evaluation of X-ray Analysis

**Figure 3:**
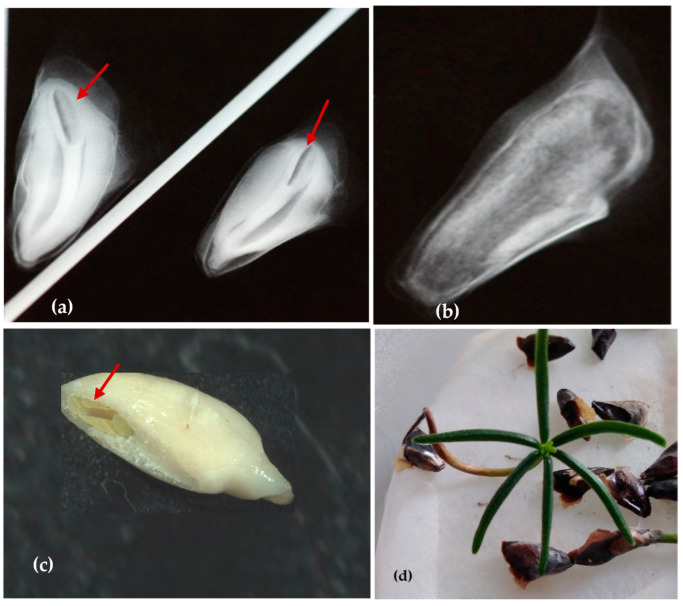
Seeds after X-ray exposure: full seed with embryo ((**a**); red arrow), empty seed (**b**) and validation of X-ray analysis: presence of embryo ((**c**); red arrow), and seed germination (**d**).

Based on the X-ray image, two groups of seeds were distinguished: (1) viable and able to germinate (Figure 3a), and (2) unviable, either empty or with an undeveloped embryo and endosperm (Figure 3b). It was observed that a significant proportion of the seeds, varying by *A. nebrodensis* tree, were devoid of ZEs [2]. The validation of X-ray analysis was confirmed through the dissection and direct observation of the X-rayed seeds, along with an assessment of their germination potential (Figure 3c,d).

The X-ray technique was essential for verifying the presence of the ZEs within the seeds [44]. This approach allowed us to save time in the excision of the ZEs used for cryopreservation purposes and EC induction, as empty seeds were discarded. Earlier, Fedorkov [45] confirmed the advantage of this method to optimize seed storage and improve the production of plant material. Although radiographic inspection is potentially harmful, seeds are exposed to a non-lethal dose during the test; hence, no damage was expected to occur or adversely affect germination [46]. 

Furthermore, X-ray analysis is a non-destructive method that safely allows the selection of high-quality seeds from deteriorating and dead seeds [47]. Therefore, X-ray tests have been widely applied to evaluate the quality of conifer seeds, such as in *Cupressus sempervirens*, *C. arizonica* [48], *Pinus sylvestris* [49,50,51], *P. sibirica*, and *P. koraiensis* [52] to separate the empty seeds from seed samples. 

### 3.2. Conservation in Liquid Nitrogen (LN, −196 °C)

#### 3.2.1. Pollen

##### Morphological Characteristics of Pollen 

**Figure 4:**
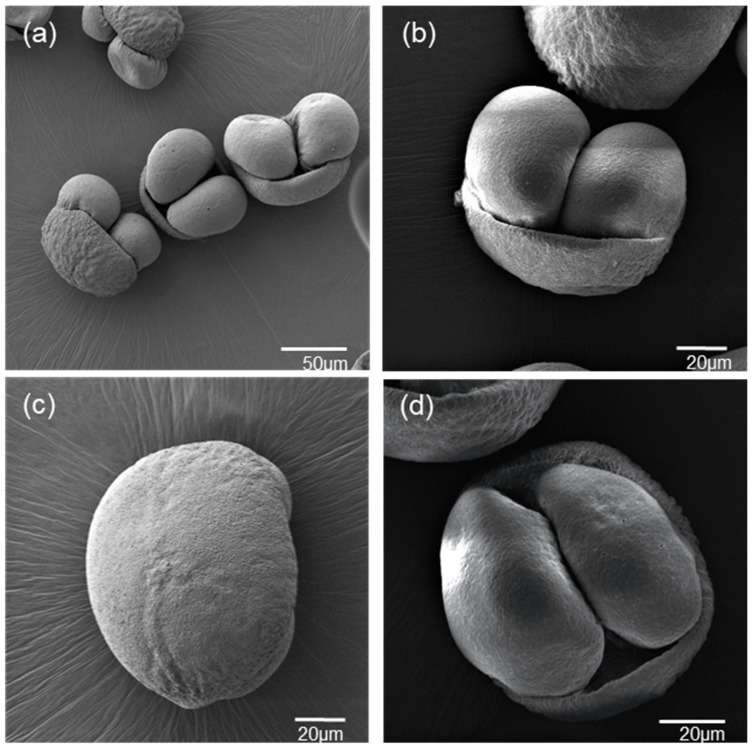
Pollen grains of *Abies nebrodensis* under SEM. (**a**) Pollen grains in the polar and equatorial views; (**b**) two sacci in equatorial views; (**c**) two sacci in polar distal view; (**d**) polar proximal view.

**Figure 5:**
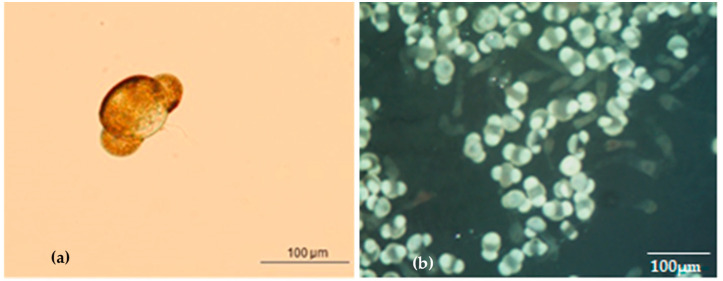
Pollen grain morphology of *A. nebrodensis* observed under optical microscope ((**a**); from Frascella et al. [1]), stereomicroscope (**b**).

The pollen of *A. nebrodensis* was observed by a scanning electron microscope (Figure 4), a Leica stereomicroscope, and a Leica Optical Microscope (Figure 5), and these examinations revealed that the pollen had the typical characteristics of the genus *Abies* [53,54,55]. Pollen grains resulted isodiametric with an elliptical central body with two lateral air sacs (bisaccate) and one aperture leptoma. The air sacs are clearly protruding from the body.

Measurements of the polar axis (P) of *A. nebrodensis* pollen grains exhibited a range from 68.11 to 92.34 µm, with an average of 83.47 µm in accordance with the study of Wrońska-Pilarek et al. [55] on *A. alba*. Most of the observed pollen grains showed a large size, over 80 µm (73%). 

##### Evaluation of Pollen MC 

**Figure 6:**
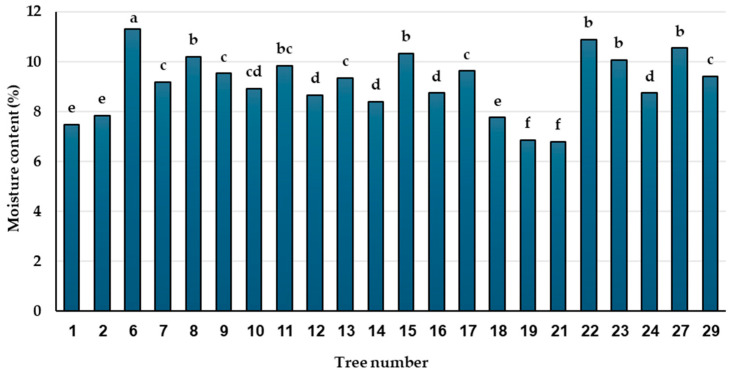
Moisture content of pollen in tested trees of *A. nebrodensis* before cryopreservation. Values with the same letters are not significantly different at the 0.05 level (Duncan’s multiple range test).

The MC in explants used in cryopreservation is a crucial factor for the success of this conservation method. For this reason, before the cryopreservation process, the MC content of pollen samples from different *A. nebrodensis* trees was recorded. Figure 6 shows that MC significantly varied among trees and was restricted in a percentage ranging between 6.8 and 11.3%. Trees 19 and 21 had the lowest percentage, while tree 6 exhibited the highest MC. 

Many factors influence the viability and germinability of cryopreserved pollen, mainly the MC, which is critical to avoid the formation of deleterious ice crystals breaking the cell membranes [12] and also the developmental stage of pollen grains (bi- or tricellular) [25,56,57].

Different methods are applied for pollen dehydration to adjust the moisture content [58,59,60]. The pollen MC from 8 to 10% avoids tissue damage during the freezing process, regardless of the final cold storage method [61]. Connor and Towill [62] reported the success of long-term conservation of pollen with moisture contents between 7 and 20% by applying −80 to −196 °C. Moreover, in some conifer species, MC percentages of pollen of 9.8–10.1 were recorded before immersion in LN [63]. In the case of *A. nebrodensis*, the MC of pollen maintained for two days at room temperature was suitable (about 10%) for direct immersion in LN without compromising its viability and germinability. This aspect is notable because it makes the cryopreservation procedure easier and faster. 

Weatherhead et al. [64] obtained successful pollen cryopreservation of potato (*Solanum* spp.) without desiccation for 9 months, with no significant reduction in pollen viability. Moreover, Anushma et al. [65] immersed the pollen of nine wild *Solanum* spp. directly in LN without dehydration; pollen viability and germinability were retained in all species without any significant reduction during the cryostorage for 36 weeks. Similarly, the cryopreservation of non-dehydrated pollen from *Diospyros* spp. and *Olea europaea* maintained both viability and germination capability for up to 360 days of storage in LN [66,67].

##### Viability and In Vitro Pollen Germinability 

**Figure 7:**
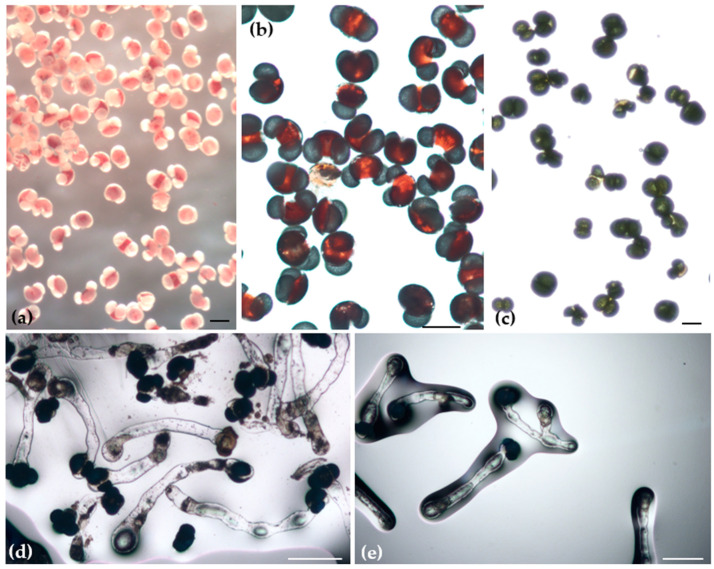
Cryopreserved pollen grains of *Abies nebrodensis*: viable pollen grains after TTC under stereomicroscope (**a**) and under microscope (**b**); non-viable pollen grains under optical microscope (**c**); in vitro germination of pollen grains under optical microscope (**d**,**e**) (Bars, 100 µm).

Pollen samples from different trees of *A. nebrodensis* were subjected to a TTC test to assess their viability in fresh and cryopreserved samples (Figure 7a–c). Results of the TTC test showed (Table 1) that pollen was viable both in the control group (−LN) and in the group that was stored in LN. Significant differences were observed in terms of tree and treatment, and the interaction between tree and treatment was also significant. In the control, the highest viability rates were recorded for trees N° 8 and 23 (98.88%), while after LN treatment, the highest viability was observed in trees 6 (95.08%) and 18 (95.52%). Trees N° 2, 19, 21, and 29 exhibited low viability rates in both the control (from 3.88 to 9.26%) and cryopreserved pollen (from 6.45 to 33.29%). However, the pollen from all trees responded favorably to the application of LN in terms of viability. The effect of the *A. nebrodensis* tree on pollen viability, both before and after cryopreservation, was significant. The highest mean viability, regardless of control and LN treatments, was recorded in trees N° 8, 17, 18, and 24, ranging from 93% to 95% (Table 1).

For the in vitro germinability test, the control and cryopreserved pollen samples (Figure 7d,e) were placed on semisolid medium and incubated at a constant temperature of 25 °C for 48 h. Observations conducted post-incubation identified the presence of pollen tube formation in both groups. The in vitro germinability outcomes indicated that the tree, treatment, and the interaction between the tree and treatment were significant (Table 2). In the control group (−LN), the germinability rate for the tree-by-treatment interaction varied from a low of 9.16% to a high of 99.72%. The highest germinability rate in the control was observed in trees N°15 (99.72%) and 14 (99.44%). Following immersion in LN, the germination capacity of the pollen from the twenty-two trees ranged from 12.66 to 99.33%. Trees N°23 and 24 showed the highest germinability, with 99.33% and 99%, respectively, whereas no germinability was observed in the cryopreserved pollen of tree 19. Generally, the treatment with LN was found to enhance pollen germination (mean 81.95%) compared to the control (mean 79.16%).

**Figure 8:**
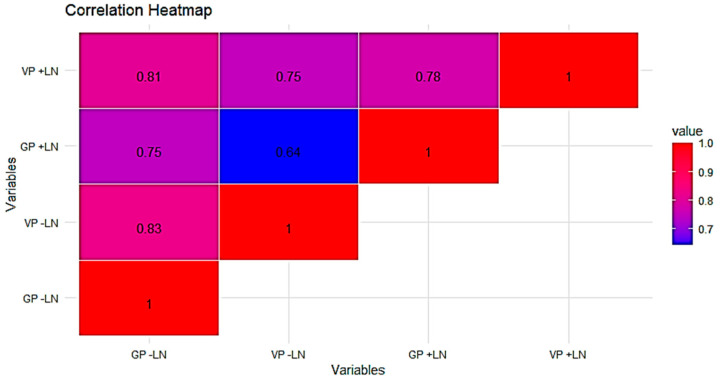
Heatmap for Pearson’s linear correlation coefficients between viability (VP) and germinability (GP) of pollen. −LN: control; +LN: immersion in liquid nitrogen.

The results of the correlation analysis revealed a significant correlation between the viability and germinability percentages of pollen before and after liquid nitrogen treatment (Figure 8). This correlation was positive either in pollen cryopreserved or not cryopreserved reporting values of R^2^ = 0.78 and R^2^ = 0.83, respectively. 

##### Pollen Catalase and Ascorbate Peroxidase Activities

**Figure 9:**
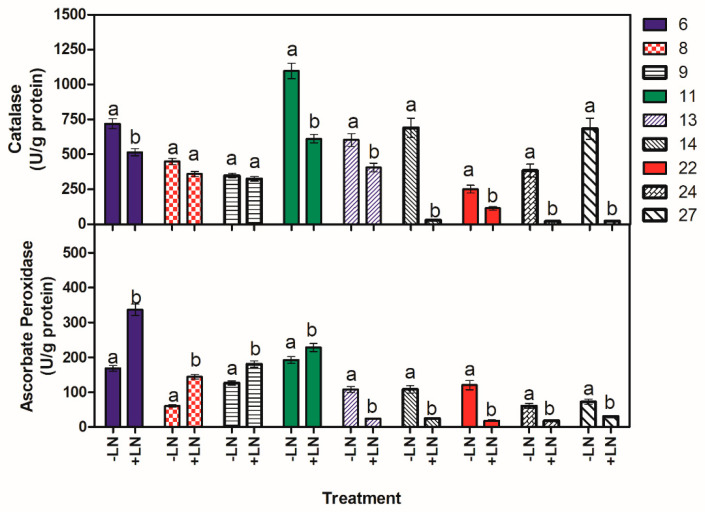
Changes of CAT and APX activity before (−LN) and after liquid nitrogen (+LN) in pollen from the tested *A. nebrodensis* trees. Different letters indicate significant differences between the values for each tree. The data are the means of three replicates.

The effects of storage temperature of LN on enzymatic activities, CAT and APX, were determined for pollen samples of nine trees (Figure 9). Changes in CAT and APX activity after freezing were different among trees. Indeed, freezing induced a reduction in CAT activity in all the trees compared with the activity measured in crude extracts from fresh pollen, with the exception of trees N°8 and 9, where this reduction was not significant. In trees 14, 24, and 27, a drastic decline was detected, with activity almost absent (about 98% of the decrease), while for the other trees, a reduction of 20–55% was observed.

Concerning the APX activity, in cryopreserved pollen of five trees out of nine examined (13, 14, 22, 24, and 27), the values decreased, and the differences compared to fresh pollen resulted in a range of 24% to 79%. The major reduction in activity was detected in the range of 70–79% for the trees N°13, 14, 22, and 24, while it was 58% for the trees N°27. In the other trees, the APX activity, after LN, significantly increased to 18% and 43% in trees 11 and 9, respectively, and almost doubled in trees 6 and 8. Changes in antioxidant enzyme activities before and after LN were significantly different for each tree, except for CAT in trees 9 and 8. Interestingly, the trees showing an increase in APX activity after LN (6, 8, 9, and 11) had the lowest decrease in CAT activity, indicating that in these trees the stress due to freezing was less damaging.

Similar to other environmental stresses, freezing and cold stress can affect plant metabolism, causing the generation of several kinds of active oxygen molecules. In response to oxidative stress, plants develop antioxidant defense systems via enzymatic and non-enzymatic antioxidant reactions to maintain normal metabolisms and functions in the cell, thus protecting themselves against oxidative stress [37,68,69,70,71]. 

CAT and APX are two significant antioxidant enzymes. Among the tested trees in this study, only three (14, 24, and 27) showed a strong decrease in CAT activity after immersion in LN. In general, an increase in antioxidant activities is normally promoted under stressful conditions to react to the stress, generating free radicals. However, according to the conditions, the response to stress can be different. The decrease in CAT activity after freezing has already been observed in frozen apple tissue [72]. This decline was attributed to damage occurring at the tetrameric enzyme structure and to the loss of the total CAT protein amount. Other findings reported that the decrease in CAT activity may be linked to the conversion of the superoxide radical to hydrogen peroxide, resulting in excessive oxidative stress [73]. Indeed, Jia et al. [74] and Jiang et al. [75] reported that the application of CAT to the LN-stored pollen could significantly improve the viability of pollen after cryopreservation. 

However, it is important to note that a considerable APX activity was present in the above-mentioned trees; it may indicate a positive response to freezing stress. 

The CAT activity declined in the most cryopreserved trees, while N°6, 8, 9, and 11 showed higher levels of both antioxidant enzyme activity (CAT and APX), and they could be considered more tolerant to the freezing condition. Despite the freezing stress, the cryopreserved pollen in the current study responded well in terms of viability and germinability, proving to be an adequate propagule for the cryopreservation of *A. nebrodensis*.

The importance of pollen cryopreservation for the long-term conservation of genetic resources has been highlighted in many plant species, including ornamental, fruit, and forest [76,77,78,79]. Pollen grains are suitable genetic materials for preservation for their small size and desiccation tolerance, and their conservation is beneficial in breeding programs overcoming seasonal and geographical restrictions [25]. Pollen from *Prunus persica* [80] and *Carya illinoensis* [81] was preserved in LN for 10 years and for 5 years in *Vitis vinifera* [82]. Zhang et al. [83] successfully cryopreserved pollen grains from 51 cultivars of *Prunus mume* for the establishment of the cryobank.

In *A. nebrodensis*, pollen preservation is fundamental to carrying out controlled cross-breeding among trees of this species to counteract the high level of inbreeding due to fragmentation and cross-contamination with other fir species present near its natural area, such as *A. alba* and *A. cephalonica.*

Pollen from conifer trees for germplasm conservation has been successfully preserved in LN. Dehydrated pollen of *Picea pungens* and *Pinus ponderosa* showed germination rates of 84% after 6 months of cryopreservation [59,62]; while for *Pseudotsuga menziesii*, the pollen cryopreserved for 1 year showed a germination rate of 81%, exhibiting a fertility nearly as high as that of fresh pollen when applied for controlled pollinations [84]. These data were in accordance with our findings on *A. nebrodensis*, where the maximum pollen germinability was recorded at 99% after cryopreservation. Lanteri et al. [63] reported good results in terms of pollen germinability after preservation at −196 °C for *Picea abies* (78.2%) and *Pinus nigra* (65.8%), without significant differences between cryopreserved and fresh pollen. In the same study, all the assessed conifer species responded well to the cryopreservation process, and it was notable that in *Picea abies*, *P. sylvestris*, and *P. uncinata*, the pollen germinability rate increased in the first month of LN storage.

In tropical forest trees, pollen cryopreservation was also reported for teak and sandalwood [85,86]. In the former species (*Tectona grandis*), after 2 days of cryopreservation, the pollen showed a very slight decrease in germination (52.18%) compared to the fresh pollen (58.25%), while in *Santalum album* L, where dried anthers with pollen were preserved in LN, no reduction was observed in the germination of cryopreserved pollen (84.20%) compared to fresh dehydrated pollen (84.50%).

The pollen viability and germinability evaluation is an essential step in verifying the success of its storage conditions. In several cryopreserved *A. nebrodensis* trees, the viability and germinability increased with respect to the control. This event was reported for other species such as *Prunus nume* [83], pineapple [78], *Solanum* spp. [65], pecan [87], mango, and litchi pollen [88]. This increase can be attributed to the very low temperature or/and the dehydration that, in combination or individually, contributed to the breakdown of the grain dormancy with an improvement in the percentage of vitality and germination [78,83]. Moreover, the viability and germinability of pollen before and after LN can be dependent on other factors such as initial grain water content and reserve compounds, external temperature, relative humidity, and gamete maturity. Indeed, Anushma et al. [65] reported that the variable response to cryopreservation observed in wild species of *Solanum* pollen could be due to inherent genetic variations and differences in the level of maturity. In Serbian spruce (*Picea omorika*), Batos and Miljkovic [89] indicated that climate conditions preceding pollen maturation influenced the pollen quality and vitality after conservation at −20 °C for 14 years. Pollen production and its subsequent performance depend on various factors, with wide variability between and within species. In particular, environmental conditions can influence pollen physiological processes such as carbohydrate storage reserves [90]. Indeed, carbohydrates, water content, and pollen viability and longevity are closely related [91,92]. In *A. nebrodensis*, the different conditions in the location of the plants (soil, wind exposure, altitudes) could influence the germination of pollen storage, even if our findings demonstrated a clear effect of the genotype.

#### 3.2.2. Zygotic Embryos 

##### Viability and In Vitro Germination of ZEs

**Figure 10:**
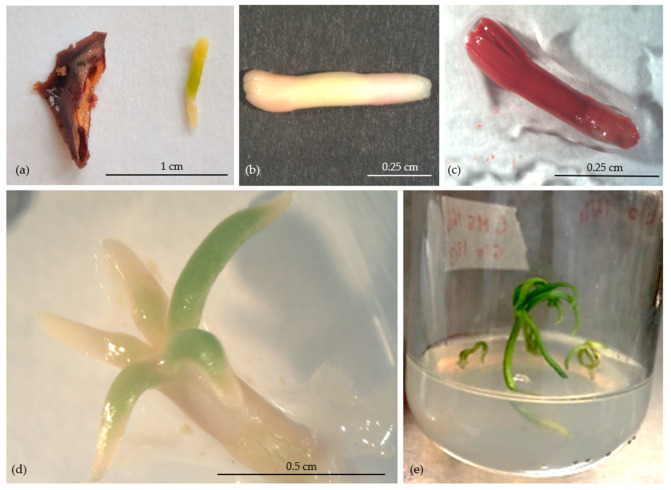
Untreated zygotic embryo (**a**), cryopreserved zygotic embryo after TTC test: non-viable (**b**) and viable (**c**); cryopreserved germinated zygotic embryo after 7 (**d**) and 20 days (**e**).

The applicability of a simplified cryopreservation protocol, with or without PVS2, for the long-term storage of ZEs was investigated for *A. nebrodensis*, and viability and germination tests were applied. Prior to cryopreservation, the MC of the non-dehydrated ZEs (Figure 10a) was 58% and decreased under the sterile laminar airflow to achieve 25–27% after 2 h [39].

The results of the viability test (Figure 10b,c) revealed that the treatment and the interaction between treatment and tree were not statistically significant (Table 3). However, the differences in tree averages were found to be statistically significant. Notably, after cryopreservation, trees N°8, 10, 13, and 27 exhibited 100% viability in the group of + PVS2 treatment. The presence of PVS2 has a protective role on plant tissues [13,27,40]. Moreover, it was interesting that only ZEs from tree N°10 maintained 100% viability after LN application, both with PVS2 and without PVS2, compared with the control. In the group −PVS2+LN treatment, viability rates of 100% were observed only in the ZEs of trees N° 6 and 10. Considering either the groups (+PVS2+LN or −PVS2+LN) the viability fluctuated between 66% and 100%. 

Table 3 demonstrated that in the control group, the percentage of viability (75.69%) was lower than those observed in both the −PVS2+LN (81.94%) and +PVS2+LN (92.01%) treatments. 

The investigation further demonstrated that the viability rates were influenced by the tree effect, as statistical analysis showed a significant variation among *A. nebrodensis* trees. These findings underscore the critical role of genetic factors in determining the susceptibility of ZEs to the cryopreservation process.

The in vitro germination test, conducted on ZEs (Figure 10d,e), revealed that the tree, treatment, and tree-treatment interaction were all statistically significant (Table 4). Germination rates exhibited variation among trees across the control group, as well as after LN applications, both with and without the submerge in PVS2. In particular, tree N°10 achieved a 100% germination rate across all three treatment conditions (control, −PVS2+LN, and +PVS2+LN). Generally, a significant reduction in germination rates for ZEs of all other trees was observed post-cryopreservation. In tree N°6, the control recorded a higher germination rate (88.89%), while, following LN treatment, the germination decreased to 16.66% without PVS2 or to 0% with PVS2. Also, the germination of trees N° 12 and 27 was null following the LN application with PVS2. These results highlight that the ZEs of the control group exhibited superior in vitro germination rates compared to the ZEs cryopreserved.

Additionally, the tree was shown to play a significant role in in vitro germination (Table 4). Tree N°10 showed the highest germination response, while in the other trees, it ranged from 14.81 to 50.37%. This underscores the significance of trees effects on the success of in vitro germination in pre- and post-cryopreservation.

**Figure 11:**
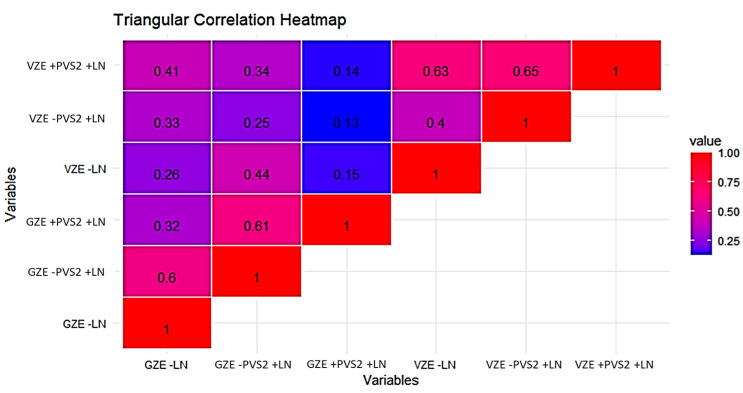
Heatmap for Pearson’s linear correlation coefficients between viability (VZE) and germination (GZE) of zygotic embryos. −LN: control; +PVS2+LN: with PVS2 and immersion in liquid nitrogen; −PVS2+LN: without PVS2 and immersion in liquid nitrogen.

A correlation analysis was conducted to explore the relationship between viability and germination in ZEs (Figure 11). The analysis revealed a non-significant correlation between viability and germination across all treatments. This finding suggests an absence of a relation between the germination and viability of ZEs, both before and after cryopreservation treatments, indicating that the processes influencing viability may not directly correspond to those affecting the germination of ZEs in the context of cryopreservation.

In conifers, Ayala et al. [39] investigated the cryopreservation of mature ZEs from the interspecific hybrid *Pinus elliottii* var. elliottii x *Pinus caribaea* var. hondurensis, achieving a 100% survival rate after 120 min of desiccation, leading to a moisture content of 23.7%. This precise desiccation treatment facilitated a high regeneration rate, with 86.7% of the surviving explants producing an average of 16.1 adventitious buds. This success, achieved with a simple desiccation step followed by direct immersion in LN, provides valuable insights into effective cryopreservation strategies. This finding is in accordance with our results, where ZEs were only desiccated and not immersed in PVS2 (−PVS2) before cryopreservation. In *Castanea sativa*, Corredoira et al. [93] reported a high survival rate of 93–100% in the embryonic axes after desiccation in a laminar flow cabinet to 20–24% MC. Following the LN storage, about 63% of these axes developed into whole plants. For the cryopreservation of embryonic axes of several *Quercus* spp. by the drying method, a recovery range of 10–80% was reported, while the treatment with 15% dimethyl sulfoxide (DMSO) solution or the application of the encapsulation-dehydration method resulted in a recovery of 40% and 12%, respectively [94]. In another study on *Citrus x aurantiifolia*, excised embryonic axes showed survival rates of 60% to 67% when undergoing dehydration and LN, significantly higher than the 10% to 13% survival following cryopreservation-vitrification with PVS2 [95]. Our investigation revealed a wide range of germination rates (0 to 100%) in post-cryopreservation, with a significant treatment-x-tree interaction, regardless of the presence of PVS2. The significant variability among trees observed in *A. nebrodensis* for some trees, demonstrating exceptionally high viability and germination rates, reflected the importance of genetic factors (the genotype effect) in determining cryopreservation success. Thus, the current cryopreservation protocol may offer a better protective effect for ZEs in some trees. Likewise, in ZEs of oil palm, a high success rate of cryopreservation was obtained, achieving up to 96.67% viability and 90.88% germination through an easy air-drying procedure without chemical pretreatment [96]. Pinto et al. [97] demonstrated that dehydration to 23% of MC achieved after 60 min, followed by osmotic solution rehydration, was optimal for cryopreservation of *Coffea arabica* ZEs, with a significant increase in germination rates. Moreover, the dehydration time significantly influenced both the viability and germination percentage of coffee ZEs, highlighting the delicate balance between removing sufficient moisture to prevent ice crystal formation and retaining enough to ensure cellular integrity. In this study, the optimal ZEs desiccation (25–27%) was obtained after 120 min under laminar airflow. 

Therefore, dehydration of ZEs prior to their introduction into LN is a method chosen for the cryopreservation of species producing recalcitrant seeds [98]. Recently, an innovative approach was applied to cryopreserve the embryonic axes of *Syzygium maire*, a species challenged by recalcitrant seeds, using a metal-mesh vacuum infiltration method with PVS2, resulting in a post-cryopreservation survival of 19% following a 20-min PVS2 incubation [99]. The difference in recovery rates among different studies highlights the species-specific and technique-specific challenges and successes inherent to cryopreservation efforts.

#### 3.2.3. Embryogenic Callus 

The capability to induce EC from mature embryos of *A. nebrodensis* was demonstrated by Jouini et al. [2], and it could be a crucial step towards ex situ conservation and large-scale propagation of this endangered fir, although it was not possible to induce EC from all the fertile trees. Indeed, the ECs were obtained from a few trees, namely N°7, 8, 10, 21, and 22, due to the variation in the response of mature ZEs to the induction medium, and even when ECs were produced, their growth was very slow. 

**Figure 12:**
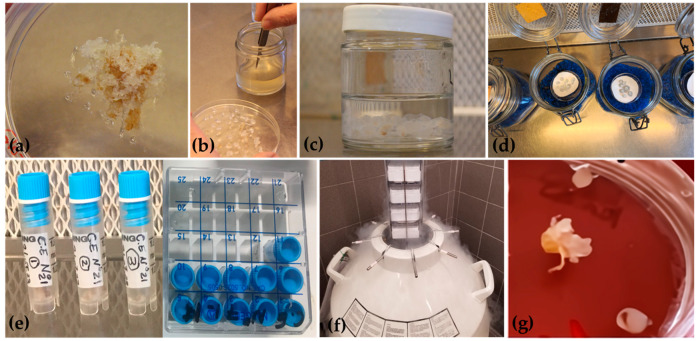
Flowchart depicts the major steps of cryopreservation for embryogenic callus of *A. nebrodensis*. Embryogenic callus-EC (**a**); preparation of EC beads (**b**,**c**); EC beads dehydration on silica gel (**d**); dehydrated EC beads in cryovials and cryoboxes (**e**); immersion in liquid nitrogen (**f**); regrowth of cryopreserved EC (**g**).

Immature or mature embryos were widely employed to establish embryogenic cultures from coniferous species [100]. To reduce costs and time spent on periodical subculture and to minimize the occurrence of somaclonal variation, somatic embryogenesis combined with cryopreservation is needed to maintain the juvenility and regenerability of lines [101]. In this study, the possibility of cryopreserving EC obtained from ZEs was investigated by applying a procedure of encapsulation-dehydration of callus samples (Figure 12). 

**Figure 13:**
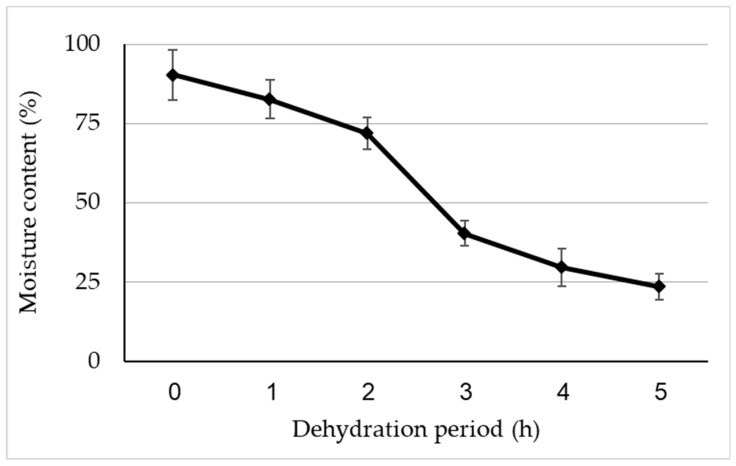
Moisture content of *A. nebrodensis* embryogenic callus beads after silica gel dehydration at different times.

Before freezing in LN, encapsulated EC beads were gradually dehydrated over silica gel. The initial MC of non-dehydrated beads was 91%, and after 5 h of desiccation, it decreased to 23% (Figure 13). This percentage was suitable for the cryopreservation of EC beads from tree N°10. Generally, the most preferred MC for encapsulated explants, either dehydrated under a laminar airflow cabinet or over silica gel, is around 20% on a fresh weight basis [27]. The optimal MC of alginate beads, prior to direct immersion in LN, is largely dependent on the plant species, e.g., 39.50% for *Phoenix dactylifera* [102], 35% for *Quercus suber* [103] and 22% for *Thymus moroderi* [104]. For *A. nebrodensis*, acceptable survival was observed for EC alginate beads with a MC of 23%. The first evidence of regrowth was visible after 4 weeks on SH medium, with 35% of EC beads developing after 8 weeks. Then, following the same procedure of encapsulation-dehydration, ECs obtained from the other lines were cryopreserved, reaching a regrowth rate of 35% in tree N°7, 30% in N°8, 32% in N°21, and 40% in N°22 by the end of 8 weeks. The recovery period, including the latency phase, is affected by the *A. nebrodensis* tree effect [105]. In our protocol, with the application of the encapsulation-dehydration technique, no cryoprotectant was used [100] to minimize the water content of calli beads, thus avoiding the toxic effect of cryoprotectants such as PVS2 [106] and lessening the risk of tissue damage.

The regeneration percentage in different conifer species determined the success of cryopreservation protocols. In the case of *A. nebrodensis*, with the encapsulation-vitrification method, 80% of recovery was achieved from non-cryopreserved EC beads, while no proliferation was obtained from those immersed in LN [2]. Our results exhibited for the first time the possibility to reach 30–40% of regrowth following the encapsulation-dehydration method after the freezing in LN, although the results depend strictly on the *A. nebrodensis* tree. Application of the slow-freezing cryopreservation protocol in hybrid firs *Abies alba × A. cephalonica* and *Abies alba × A. numidica* led to 37.5%–100% regrowth of cryopreserved embryogenic tissues [33]. Similarly, applying the same method to embryogenic lines from *A. alba* enabled regeneration ranging from 91.66 to 100% [107]. Slow-freezing is an effective method for the cryopreservation of EC, but it is not simple and requires specific programmable apparatus for slow-cooling before the sample immersion into LN. A proper protocol with an 80% survival by vitrification method was optimized to cryopreserve embryogenic cell lines of *Larix kaempefri × Larix gmelinii*, involving pre-culturing of embryogenic tissue on a medium containing an osmotic agent, followed by cryoprotectant treatment [108].

Survival rates of 84.4% and 86.7% were reached by the stepwise dehydration method (sucrose steps from 0.25 to 1.0 M for 7 days, followed by desiccation over silica gel) of embryogenic tissues from *A. alba* × *A. numidica* and *P. nigra*, respectively, 28 and 35 days after recovery from LN [109]. In another study with the vitrification method, Latutrie and Aronen [110] observed 80–93% recovery for *P. sylvestris* embryogenic lines. 

Regrowth with our encapsulation-dehydration protocol was reliable but lower than for other conifer species, regardless of cryopreservation technique. The preliminary results obtained from the current study demonstrate that the encapsulation-dehydration method can be successfully used for the cryopreservation of *A. nebrodensis* ECs. Further investigation is needed to improve the regrowth percentage after immersion in LN of EC of Sicilian fir, such as pre-culture of encapsulated ECs in medium supplemented with osmotic agents for a specific time or modification of the regrowth medium to optimize the recovery percentage. To the best of our knowledge, this is the first report on the application of this technique for *A. nebrodensis*, offering an alternative method for the efficient and long-term storage of its germplasm in LN.

## 4. Conclusions

This study, developed in the framework of the LIFE4FIR Project, financed by the European Union, represents a qualifying example of actions aimed at safeguarding the Sicilian fir, which is listed by the International Union for Conservation of Nature (IUCN) as an endangered species due to serious genetic erosion. For this reason, the plan of protection of the 30 relict trees involved a series of actions combining two strategies: in situ, by monitoring their health status and protecting their survival in the natural habitat, and *ex situ*, through the conservation of the seeds and the cryopreservation of organs and tissues taken from the relict trees. *A. nebrodensis* is a conifer characterized by the presence of a low percentage of full seeds with an unknown life span in terms of years, even in the sporadic years of abundant seed production. Therefore, in addition to the establishment of a ‘Seed Bank’, the project, through a cryopreservation approach, has obtained effective conservation of pollen, zygotic embryos, and lines of embryogenic callus in liquid nitrogen. In this study, the cryopreservation protocols for several explants of *A. nebrodensis* were optimized and validated, reaching a satisfactory recovery percentage of pollen, ZEs, and EC after LN. Therefore, the obtained results enabled the secure preservation of the germplasm of the remaining population and the establishment of a cryobank inside the ‘MAN/Museum of the *A. nebrodensis*’ located near the ‘Madonie Regional Park’ in the Municipality of Polizzi Generosa. 

To our knowledge, the Sicilian fir ‘Cryobank’ is currently a unique example for the application of cryopreservation technique to a conifer at immediate risk of extinction, can pave the way for similar strategies with other conifers subjected to advanced genetic erosion, and also serves as a critical model to preserve biodiversity in facing the challenges of environmental changes. 

## Figures and Tables

**Table 1:** Viability percentage of pollen before and after cryopreservation.

N° Tree (T)	Treatment (Tr)	Tr xT	T Mean	Tr Mean
1	Control	77.77 h–l	76.14 GHI	
	+LN	74.51 i–m		
2	Control	9.16 n	7.81 LM	
	+LN	6.45 n		
6	Control	90.00 b–f	92.54 ABC	
	+LN	95.08 abc		
7	Control	88.89 c–g	79.51 FGH	
	+LN	70.14 klm		
8	Control	98.88 a	95.29 A	
	+LN	91.69 b–f		
9	Control	79.16 g–k	85.94 DEF	
	+LN	92.71 bcde		
10	Control	88.16 b–f	90.94 ABCD	
	+LN	93.72 bcd		
11	Control	90.55 b–f	92.10 ABCD	
	+LN	93.66 bcd		Control
12	Control	65.88 lm	74.08 GHI	68.93 B
	+LN	82.27 f–j		
13	Control	90.44 bcde	89.89 BCD	
	+LN	89.33 b–g		+LN
14	Control	74.99 i–m	74.07 HI	73.22 A
	+LN	73.14 i–m		
15	Control	65.83 lm	68.27 IJ	
	+LN	70.70 klm		
16	Control	90.27 b–f	87.14 DEF	
	+LN	84.02 e–i		
17	Control	93.61 bcd	93.80 AB	
	+LN	93.99 bcd		
18	Control	96.39 ab	95.95 AB	
	+LN	95.52 abc		
19	Control	3.88 n	5.79 M	
	+LN	7.69 n		
21	Control	4.99 n	34.68 K	
	+LN	33.29 m		
22	Control	33.05 m	59.85 J	
	+LN	86.65 d–h		
23	Control	98.88 a	82.63 CDE	
	+LN	66.38 lm		
24	Control	94.44 bcd	93.86 AB	
	+LN	93.28 bcd		
27	Control	71.94 jklm	82.09 EFG	
	+LN	92.24 b–f		
29	Control	9.26 n	16.85 L	
	+LN	24.43 m		
*p* value		0.0002 ***	0.0001 ***	0.0001 ***

LSD_tree_: 5.81 ***. LSD_treatment_: 1.75 ***. LSD_tree×treatment_: 8.22 ***. *** *p* < 0.001; +LN: Immersed in liquid nitrogen; −LN: Control not immersed in liquid nitrogen. The values of Tr xT are the mean of two replicates/tree (at least 300 pollen grains/replicate). Different letters within a column indicate significantly different means (LSD test). Lowercase letters indicate significant differences between *A. nebrodensis* trees and treatments. Uppercase letters indicate significant differences among total trees and the mean of treatments.

**Table 2:** Germinability percentage of pollen before and after cryopreservation.

N° Tree (T)	Treatment (Tr)	TrxT	T Mean	Tr Mean
1	Control	84.72 l–p	83.69 G	
	+LN	82.66 op		
2	Control	22.62 qr	20.83 I	
	+LN	41.66 q		
6	Control	88.89 i–p	87.77 EFG	
	+LN	86.66 k–p		
7	Control	90.00 h–p	85.00 FG	
	+LN	80.00 p		
8	Control	91.11 h–p	91.55 DEF	
	+LN	92.00 g–o		
9	Control	87.77 j–p	88.38 EFG	
	+LN	89.00 i–p		
10	Control	98.61 abcd	95.80 ABC	
	+LN	93.00 f–o		
11	Control	93.89 d–l	92.44 CDE	
	+LN	91.00 h–p		
12	Control	92.22 g–o	91.44 CDE	Control
	+LN	90.66 e–m		79.16 B
13	Control	89.44 i–p	92.88 CDE	
	+LN	96.33 a–h		+LN
14	Control	99.44 ab	95.38 ABC	81.95 A
	+LN	91.33 e–n		
15	Control	99.72 a	96.69 AB	
	+LN	93.66 b–j		
16	Control	90.83 h–p	94.25 BCD	
	+LN	97.66 a–g		
17	Control	85.83 l–p	92.08 CDE	
	+LN	98.33 a–e		
18	Control	83.61 n–p	84.14 FG	
	+LN	84.66 m–p		
19	Control	23.89 qr	11.94 I	
	+LN	0.00 t		
21	Control	30.28 q	62.14 H	
	+LN	94.00 c–k		
22	Control	91.66 g–o	92.50 CDE	
	+LN	93.33 e–m		
23	Control	98.05 a–f	98.69 A	
	+LN	99.33 a		
24	Control	98.89 abcd	98.94 A	
	+LN	99.00 abc		
27	Control	90.83 h–p	93.41 CDE	
	+LN	96.00 a–i		
29	Control	9.16 s	10.91 I	
	+LN	12.66 rs		
*p* value		0.0001 ***	0.0009 ***	0.0001 ***

LSD_tree_: 6.69 ***. LSD_treatment_: 2.01 ***. LSD_tree×treatment_: 9.47 ***. *** *p* < 0.001; +LN: Immersed in liquid nitrogen; −LN: Control not immersed in liquid nitrogen. The values of Tr xT are the mean of two replicates/tree (at least 300 pollen grains/replicate). Different letters within a column indicate significantly different means (LSD test). Lowercase letters indicate significant differences between *A. nebrodensis* trees and treatments. Uppercase letters indicate significant differences among total trees and the mean of treatments.

**Table 3:** Viability percentage of zygotic embyos before and after cryopreservation.

N° Tree (T)	Treatment (Tr)		TrxT	T Mean	Tr Mean
6	Control	UN	33.33 a		
	+LN	−PVS2	100 a	74.07 BC	
		+PVS2	88.88 a		
8	Control	UN	83.33 a		
	+LN	−PVS2	88.88 a	90.74 ABC	
		+PVS2	100 a		
10	Control	UN	100 a		Control
	+LN	−PVS2	100 a	100 A	75.69 A
		+PVS2	100 a		
12	Control	UN	66.66 a		
	+LN	−PVS2	66.66 a	66.66 C	
		+PVS2	66.66 a		−PVS2 +LN
13	Control	UN	88.89 a		81.94 A
	+LN	−PVS2	66.66 a	85.18 ABC	
		+PVS2	100 a		
21	Control	UN	100 a		
	+LN	−PVS2	77.77 a	88.88 ABC	+PVS2 +LN
		+PVS2	88.88 a		92.01 A
22	Control	UN	33.33 a		
	+LN	−PVS2	72.22 a	65.74 C	
		+PVS2	91.66 a		
27	Control	UN	100 a		
	+LN	−PVS2	83.33 a	94.44 AB	
		+PVS2	100 a		
*p* value			0.2503	0.0498 *	0.1126

LSD_tree_: 22.91 *. LSD_treatment_: Not Significant. LSD_tree×treatment_: Not Significant. * *p* < 0.05. UN: untreated Control, neither PVS2 nor LN. The values of Tr xT are the mean of three replicates per tree (6 zygotic embryos/replicate). Different letters within a column indicate significantly different means (LSD test). Lowercase letters indicate significant differences between *A. nebrodensis* trees and treatments. Uppercase letters indicate significant differences among total trees and the mean of treatments.

**Table 4:** Germination percentage of zygotic embryos before and after cryopreservation.

N° Tree (T)	Treatment (Tr)		TrxT	T Mean	T Tr Mean
6	Control	UN	88.89 ab		
	+LN	−PVS2	16.66 fghi	35.18 B	
		+PVS2	0.00 i		
8	Control	UN	83.33 abc		
	+LN	−PVS2	58.33 cde	50.00 B	
		+PVS2	8.33 ghi		
10	Control	UN	100 a		
	+LN	−PVS2	100 a	100 A	Control
		+PVS2	100 a		65.97 A
12	Control	UN	33.33 efgh		
	+LN	−PVS2	33.33 efgh	14.81 C	
		+PVS2	0.00 i		−PVS2 +LN
13	Control	UN	77.78 abc		39.79 B
	+LN	−PVS2	58.33 cde	50.37 B	
		+PVS2	15 fghi		
21	Control	UN	75 bcd		
	+LN	−PVS2	35 defg	46.66 B	+PVS2 +LN
		+PVS2	30 d-f		25.11 B
22	Control	UN	36.11 defg		
	+LN	−PVS2	5.55 hi	29.76 BC	
		+PVS2	47.61 cdef		
27	Control	UN	33.33 efgh		
	+LN	−PVS2	11.11 ghi	14.81 C	
		+PVS2	0.00 i		
*p* value			0.0003 ***	0.0001 ***	0.0001 ***

LSD_tree_: 15.79 ***. LSD_treatment_: 11.63 ***. LSD_tree×treatment_: 27.36 ***. *** *p* < 0.001; UN: untreated Control, neither PVS2 nor LN. The values of Tr xT are the mean of three replicates per tree (6 zygotic embryos /replicate). Different letters within a column indicate significantly different means (LSD test). Lowercase letters indicate significant differences between *A. nebrodensis* trees and treatments. Uppercase letters indicate significant differences among total trees and the mean of treatments.

## Data Availability

Data are contained within the article.
